# Supplementary material for: Examining the impact of green marketing practices on business performance: A synergistic application of resource-based view and triple bottom line theory
Source: PLoS One. 2026 Jan 20;21(1):e0333026. doi: 10.1371/journal.pone.0333026 (PMC12818679; doi:10.1371/journal.pone.0333026)
Supplement: S1 Appendix — (DOCX) [file pone.0333026.s001.docx]

**Appendix-A (Constructs and Items)**

| Variable | Statement |
| --- | --- |
| Green Marketing Practice | Our company cooperates with environmentally friendly partners. |
|  | Our company uses eco-labels on packaging. |
|  | Our company applies a paperless policy in our procurement where possible. |
|  | Our company applies a paperless policy in our personnel management where possible. |
| Perceived Competitive Advantages | Our firm can enter new, lucrative markets with the adoption of environmental strategies. |
|  | Our firm can penetrate the market by making existing goods more friendly to the environment. |
|  | By reducing the environmental impact of our firm’s activities, the quality of the products will improve. |
|  | Perceived competitive advantages being an environmentally-conscious firm can lead to cost advantages within our firm. |
| Green Innovative Marketing Strategy | We participate in environmental business networks. |
|  | We use specific environmental policy for selecting our partners. |
|  | We implement market research to detect green needs in the marketplace. |
|  | We have created a separate department/unit specializing in environmental issues for our organization. |
| Business Performance | Firm’s profitability increases due to Green Marketing Practice. |
|  | Our customer satisfaction becomes high. |
|  | Our customer becomes more loyal. |
|  | We reputation among customers and industry. |
| Environmental Performance | Our industry has reduced its carbon foot prints and greenhouse gas emissions. |
|  | Our organization established there duction of solid, liquid, and energy wastes. |
|  | Our industry has reduced the use of hazard, noxious, or toxic materials in our operations. |
|  | Our company has reduced waste a cross our process. |
| Economic Performance | Our organization reduced costs of production. |
|  | Our organization’s profit has improved. |
|  | Our organization reduced product development costs and energy consumption cost. |
|  | Our company has a program for the collection and recycling of products and materials. |
| Social Performance | Our organization improved the working environment and people’s morale. |
|  | Our organization prioritizes the health and safety of employees. |
|  | Our organization improved labor relations. |
|  | Our value chain has fair working conditions |
