# Supplementary material for: Examining the impact of green marketing practices on business performance: A synergistic application of resource-based view and triple bottom line theory
Source: PLoS One. 2026 Jan 20;21(1):e0333026. doi: 10.1371/journal.pone.0333026 (PMC12818679; doi:10.1371/journal.pone.0333026)
Supplement: Attachment — Submitted filename: PONE-D-25-48491_reviewer.docx [file pone.0333026.s002.docx]

This manuscript investigation is to assess the influence of green marketing practices and their potential effects on organizations' operational performance. While the topic is timely and practically relevant, the manuscript faces several critical issues.

1. In the introduction section, please supplement the overview of relevant research on green marketing. The manuscript only seems to extract the importance of research from the practical background, ignoring the research gap, the necessity of unanswered research questions, how the research questions are solved in the existing research, and the unsolved problems that the manuscript intends to solve from what perspective.

2. Although the manuscript mentions that "business performance is indicated by environmental, economic and social performance as per the triple bottom line (TBL) theory”, environmental, economic and social performance should be taken as the three dimensions of business performance rather than as the outcome variables of business performance.

3. In the description of the data collection section, the authors mention that " We disseminated 400 questionnaires across various enterprises (manufacturing, nonmanufacturing, and service sectors)", whether the "various enterprises" mentioned here have implemented green marketing? In addition, whether the subjects understand the enterprise performance also needs to be considered, and usually the middle and senior managers are familiar with the enterprise performance.

4. In the theoretical contribution section, it is suggested to elaborate the theoretical contribution in sections and echo the existing theories.
