# Supplementary material for: Examining the impact of green marketing practices on business performance: A synergistic application of resource-based view and triple bottom line theory
Source: PLoS One. 2026 Jan 20;21(1):e0333026. doi: 10.1371/journal.pone.0333026 (PMC12818679; doi:10.1371/journal.pone.0333026)
Supplement: Attachment — Submitted filename: Response to Reviewers.docx [file pone.0333026.s004.docx]

**Review Comments**

This manuscript investigation is to assess the influence of green marketing practices and their potential effects on organizations' operational performance. While the topic is timely and practically relevant, the manuscript faces several critical issues.

1. In the introduction section, please supplement the overview of relevant research on green marketing. The manuscript only seems to extract the importance of research from the practical background, ignoring the research gap, the necessity of unanswered research questions, how the research questions are solved in the existing research, and the unsolved problems that the manuscript intends to solve from what perspective.

**Author’s reply:**

*Thank you for this valuable comment. We have thoroughly revised the Introduction section to provide a deeper engagement with existing literature on green marketing. We have incorporated key studies that highlight prior findings, theoretical perspectives, and remaining research gaps. Specifically, we now (a) synthesize prior empirical works on green marketing practices and firm performance; (b) identify the inconsistent results regarding their direct and indirect effects; and (c) highlight how integrating the Resource-Based View (RBV) with the Triple Bottom Line (TBL) framework offers a novel perspective to explain performance outcomes across environmental, economic, and social domains.*

2. Although the manuscript mentions that "business performance is indicated by environmental, economic and social performance as per the triple bottom line (TBL) theory”, environmental, economic and social performance should be taken as the three dimensions of business performance rather than as the outcome variables of business performance.

**Author’s reply:**

*We sincerely appreciate the reviewer's insightful observation regarding the conceptual positioning of environmental, economic, and social performance within the Triple Bottom Line (TBL) framework. We agree that, according to Elkington's (1997) original formulation, these three dimensions collectively constitute the broader construct of business performance, reflecting a firm's sustainability orientation.*

*However, in the current study, we have intentionally operationalized environmental, economic, and social performance as distinct yet interrelated outcome variables of business performance to empirically capture the multidimensional manifestation of sustainability performance in line with the research objectives, which have treated these dimensions as separate dependent constructs to better understand how managerial and organizational factors drive each performance outcome differently.*

*Furthermore, our measurement model follows the reflective–formative hierarchy commonly adopted in sustainability research, in which the overall construct (business performance) is reflected through its three distinct components, environmental, economic, and social outcomes. This modeling approach allows for a more nuanced interpretation, as each dimension responds to organizational practices and strategies in different magnitudes and directions. To ensure conceptual clarity, we have slightly revised the manuscript's phrasing to emphasize that "environmental, economic, and social performance are the three outcome dimensions through which sustainable business performance is assessed, consistent with the TBL perspective." This wording acknowledges the reviewer's concern while maintaining the integrity of our empirical model.*

3. In the description of the data collection section, the authors mention that " We disseminated 400 questionnaires across various enterprises (manufacturing, nonmanufacturing, and service sectors)", whether the "various enterprises" mentioned here have implemented green marketing? In addition, whether the subjects understand the enterprise performance also needs to be considered, and usually the middle and senior managers are familiar with the enterprise performance.

**Author’s reply:**

*We are grateful to the reviewer for this valuable comment that enhances the clarity and rigor of our data collection description. We want to clarify that the enterprises included in our survey were carefully selected for their engagement in environmental or green initiatives. Specifically, prior to distributing the questionnaires, we verified through company websites, sustainability reports, and local environmental directories that these organizations had either implemented green marketing, environmental management systems, or sustainability-related practices. Thus, the sample appropriately represents enterprises that have adopted green or sustainability practices relevant to the study’s context.*

*Regarding the second concern, we fully agree that respondents’ familiarity with enterprise performance is crucial to ensure data reliability. Accordingly, our target respondents were middle- and senior-level managers, including those working in marketing, sustainability, production, and corporate strategy, who are directly involved in decision-making and possess a comprehensive understanding of both operational and performance outcomes.*

*To further address this valuable comment, we have revised the data collection section in the manuscript.*

4. In the theoretical contribution section, it is suggested to elaborate the theoretical contribution in sections and echo the existing theories.

**Author’s reply:**

*We sincerely thank the reviewer for this constructive suggestion. We fully agree that elaborating the theoretical contribution in a structured manner and linking it more explicitly to the underlying theories will enhance the manuscript’s theoretical rigor and clarity. In response, we have revised the theoretical contribution section to clearly articulate how this study advances existing theories and frameworks used in the research. [revised in yellow]*
