# Supplementary material for: Examining the impact of green marketing practices on business performance: A synergistic application of resource-based view and triple bottom line theory
Source: PLoS One. 2026 Jan 20;21(1):e0333026. doi: 10.1371/journal.pone.0333026 (PMC12818679; doi:10.1371/journal.pone.0333026)
Supplement: Attachment — Submitted filename: Response_to_Reviewers_auresp_2.docx [file pone.0333026.s005.docx]

**Review Comments (Reviewer1)**

Reviewer #1: Thanks for inviting me to evaluate Manuscript entitled “Examining the Impact of Green Marketing Practices on Business Performance: A Synergistic Application of Resource-Based View and Triple Bottom Line Theory”
This study investigates the relationship between green marketing practices (GMP) and business performance (BP), employing the Resource-Based View (RBV) and Triple Bottom Line (TBL) theories. Although I think the goal of the paper is clear and is well motivated, the manuscript could be improved from the following aspects.

***I am really grateful to the reviewer for giving us the opportunity to revise the manuscript, which will help enhance its quality.***

1.The paper operationalizes environmental, economic, and social performance as outcomes of BP. However, prior literature often treats these as dimensions of BP itself. More destailed Clarification of this distinction are needed. The authors can revise the conceptual framework to explicitly show whether BP is a higher-order construct with three dimensions or a precursor to these outcomes.

Author’s reply

Thank you for this insightful comment. We agree that much of the prior literature conceptualizes business performance as a multidimensional construct reflecting environmental, economic, and social dimensions. However, our study intentionally adopts a different theoretical stance grounded in the Resource-Based View (RBV) and the Triple Bottom Line (TBL).

Specifically, we distinguish Business Performance (BP) from the three sustainability-related outcomes. Business Performance is modeled as an antecedent, and environmental, economic, and social performance are modeled as distinct outcome constructs rather than higher order constructs. (**Added the description of the conceptual framework**)

2. While PCA and GIMS are positioned as mediators, the rationale for their selection over other potential mediators (e.g., stakeholder pressure, institutional support) is underdeveloped. The theoretical justification should be strengthened on green marketing mediators.

Author’s reply

The selection of Perceived Competitive Advantages (PCA) and Green Innovative Marketing Strategy (GIMS) as mediators is grounded in the central logic of the Resource-Based View (RBV), which emphasizes that firm performance stems from the development and deployment of internal, valuable, rare, and inimitable resources. Green marketing practices alone do not automatically create superior performance; rather, they must be transformed into internal competitive capabilities that enable firms to outperform competitors. PCA and GIMS represent precisely these capability-building mechanisms. [Literature review section revised]

3.The study claims to include firms engaged in GMP but lacks details on how green status was verified. More description about the criteria for selecting green enterprises should be added.

Author’s reply

Thank you for highlighting this important issue. We agree that the criteria used to identify and verify firms’ engagement in green marketing practices (GMP) required further clarification. In response, we have added a detailed description of the screening procedures and eligibility criteria employed during the sampling process. (**Revised**)
4. Although VIF scores (<3.3) suggest minimal CMB [9], the reliance on self-reported data from managers raises concerns about social desirability bias.

Author’s reply

We appreciate the reviewer’s insightful observation. While VIF values indicated that common method bias (CMB) was unlikely to pose a significant threat, we agree that self-reported data from managers may still be subject to social desirability bias. In response, we have added a detailed explanation of the procedural and statistical remedies employed to mitigate this concern.

Specifically, we emphasize that (1) respondents were assured of full anonymity and confidentiality, [**mentioned in methods section**] (2) neutral and non-evaluative wording was used throughout the questionnaire, (3) items measuring predictors and outcomes were placed in separate sections to reduce implicit consistency motives. These steps collectively minimized the potential inflation of relationships due to socially desirable responding.
5.The path coefficients (e.g., GMP → BP: β= 0.326; BP → ENP: β= 0.653) are statistically significant but modest. The authors should discuss practical significance to contextualize findings.

Author’s reply

Thank you for this valuable comment. We agree that statistical significance alone does not fully capture the managerial or practical relevance of the results. In response, we have added a discussion highlighting the *practical significance* of the observed effect sizes. Although some coefficients (such as GMP --> BP at β=0.326) are moderate in magnitude, they reflect meaningful real-world impacts given the complexity and multi-dimensional nature of green marketing implementation. The discussion now clarifies that incremental improvements in GMP or BP can produce substantial sustainability gains over time, especially in resource-constrained or developing-country contexts. This addition has been incorporated into the Discussion section.
6. Firm size, industry sector, and regulatory environment may confound the GMP-BP relationship.

Author’s reply

Thank you for this thoughtful comment. We agree that contextual factors such as firm size, industry type, and regulatory environment may influence both the adoption of green marketing practices and the resulting business performance. To address this, we have revised the manuscript by acknowledging these potential confounders in the limitations & future research in **conclusion** section.

While the current study did not incorporate all these variables as controls due to data constraints, we now clearly state that future research should explicitly examine and control for these contextual characteristics. This addition strengthens the transparency of our methodological approach and provides clear directions for enhancing the robustness and generalizability of future studies on green marketing and business performance.
7. In the Discussion part, the theoretical contributions is fragmented. The theoretical grounding need to be enhenced.

Author’s reply

Theoretical contribution section is revised.
